# Type IV RTA in Chronic Adrenal Insufficiency and Concomitant Lisinopril Treatment

**DOI:** 10.1155/2020/8897112

**Published:** 2020-10-19

**Authors:** Francesca Galbiati

**Affiliations:** Department of Medicine, University of Pittsburgh Medical Center, 200 Lothrop Street, Suite N715, Pittsburgh, PA 15213, USA

## Abstract

Type IV renal tubular acidosis (RTA) is the only RTA characterized by hyperkalemia, and it is caused by a true aldosterone deficiency or renal tubular aldosterone hyporesponsiveness. It is frequent among hospitalized patients as it is related to type 2 diabetes mellitus (T2DM) and common medications such as ACE-inhibitors (ACE-is) and trimethoprim-sulfamethoxazole (TMP-SMX). Drug-induced RTA commonly manifests in patients with predisposing conditions such as mild renal insufficiency and certain pharmacological therapies. ACE-i use and chronic adrenal insufficiency (cAI) are other significant risk factors. Chronic ACTH suppression is thought to induce global adrenal atrophy, including the zona glomerulosa, thus affecting aldosterone secretion as well. Furthermore, in the setting of cAI, treatment with ACE-is further suppresses aldosterone production. This case report describes a patient with cAI secondary to corticosteroid use for years who developed type IV RTA in the setting of lisinopril use. Potassium (K) elevation persisted despite removing underlying conditions and metabolic acidosis correction. The patient required long-term treatment with mineralocorticoids in addition to sodium bicarbonate to maintain normal K levels and acid-base status. Mineralocorticoid administration is a second-line treatment for type IV RTA, but it might be necessary for a subgroup of high-risk patients. In fact, it is important to consider patients with chronic adrenal insufficiency and on ACE-is treatment at increased risk for refractory hyperkalemia in the setting of type IV RTA. Indeed, this subgroup of patients can have severe hypoaldosteronism.

## 1. Introduction

**Figure 1:**
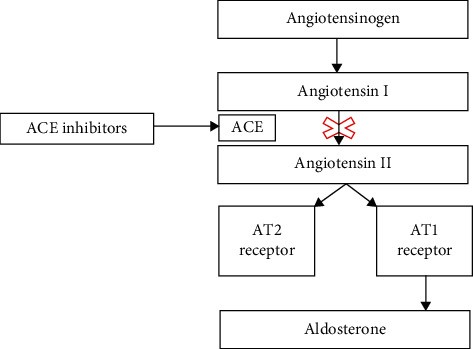
Effects of ACE-inhibitors on angiotensin conversion. ACE-inhibitors suppress the ACE enzyme action, thus blocking the conversion of angiotensin I to angiotensin II and its action on AT2 and AT1 receptors. The interaction between angiotensin II and AT1 receptors leads to aldosterone production.

Type IV renal tubular acidosis (RTA) is the only RTA characterized by hyperkalemia, and it is caused by a true aldosterone deficiency or renal tubular aldosterone hyporesponsiveness [1]. It is frequent among hospitalized patients as it is related to type 2 diabetes mellitus (T2DM) and common medications such as ACE-inhibitors (ACE-is) and trimethoprim-sulfamethoxazole (TMP-SMX), and it occurs more often in patients with chronic kidney disease (CKD) [2]. Drug-induced RTA commonly manifests in patients with predisposing conditions, for example, the use of diclofenac and mild renal insufficiency exacerbated the potassium-altering effect of trimethoprim [3]. ACE-i use ° and chronic adrenal insufficiency (cAI) are other significant risk factors. It is well known that chronic steroid treatment is a major cause of ACTH suppression [4]. Chronic ACTH suppression is thought to induce global adrenal atrophy [4, 5], including the zona glomerulosa, thus affecting aldosterone secretion as well. In cAI, treatment with ACE-is further suppresses aldosterone production by inhibiting the angiotensin-converting enzyme (Figure 1). In the clinical setting of cAI and concomitant ACE-i treatment, type IV RTA can be refractory to first-line interventions with subsequent persistent hyperkalemia requiring treatment with mineralocorticoids in addition to sodium bicarbonate. The literature on type IV RTA in patients with secondary or iatrogenic causes of hypoaldosteronism is sparse and requires more data.

## 2. Case Presentation

A 66-year-old female with a history of rheumatoid arthritis on prednisone 5 mg daily, T2DM, CKD, AI secondary to chronic steroid use, and recent hospitalization for methicillin-resistant *Staphylococcus aureus* (MRSA) osteomyelitis still on antibiotics, now admitted for cholangitis treatment, suddenly presents unexplained hyperkalemia. At the time of admission, the patient was started on piperacillin-tazobactam for cholangitis and on TMP-SFX for a history of MRSA osteomyelitis. She also received stress-dose steroids for 48 hours with immediate clinical improvement. The patient's hospitalization was prolonged (more than 30 days) and complicated by acute kidney injury, hyperkalemia, and nonanion gap metabolic acidosis (NAGMA) outlined as follows. The patient also had a history of hypertension treated with lisinopril 5 mg daily, which was initially discontinued and restarted during the hospitalization (day 30) at the increased dose of 10 mg daily given severe hypertension. On day 33, the patient's creatinine rose to 1.5 mg/dL from a baseline of 1.2 mg/dL, and her previously normal potassium (K) increased to 5.5 mmol/L (Table 1). This was thought to be related to lisinopril, which was discontinued. Off lisinopril, her kidney function recovered, but unexpectedly, hyperkalemia worsened to 6.3 mmol/L, and she presented a new-onset NAGMA with serum bicarbonate 17.5 mmol/L. The abnormally elevated K was then attributed to TMP-SMX-induced hyperkalemia, so TMP-SFX was switched to doxycycline, and the patient was given patiromer. Despite these interventions, both hyperkalemia and NAGMA persisted (K, 6.3 mmol/L and bicarbonate, 19.6 mmol/L on day 35). At that point, urine electrolytes and venous blood gas were obtained, demonstrating a positive urine anion gap and venous pH of 7.31. The urinalysis was normal with pH of 7, no evidence of infection, and absence of proteinuria, glycosuria, and microhematuria. Plasma renin activity was 0.95, and aldosterone levels were undetectable; however, the treatment with ACE-I could affect the interpretation of this test. The picture was now consistent with type IV RTA, so the patient was started on oral sodium bicarbonate 1300 mg three times a day with the resolution of NAGMA. Despite the discontinuation of possible culprit medications, the patient was still hyperkalemic. Finally, considering her cAI with a likely component of hypoaldosteronism worsened by lisinopril, fludrocortisone 0.1 mg was added daily with immediate normalization of K (from 5.2 mmol/L to 4.4 mmol/L). This happened the fourth day after stopping lisinopril (Table 1). The patient had to continue the daily therapy with both sodium bicarbonate and fludrocortisone to maintain normal K levels and acid-base status and did not experience any electrolyte imbalance or worsening hypertension, while on the mineralocorticoid replacement therapy, as a demonstration that, she was indeed aldosterone-deficient. Of note, cortisol and adrenocorticotropic hormone levels were not measured during this admission as the patient's diagnosis of adrenal insufficiency was well established, and she was on treatment.

## 3. Discussion

The classical manifestations of type IV RTA are hyperkalemia and NAGMA. In the inpatient setting, these are frequently primarily attributed to patients' comorbidities and polypharmacy, thus missing the correct diagnosis. The type IV RTA workup (serum K, urine electrolytes and anion gap, urine pH, and urine bicarbonate) is cost-effective and straightforward and should be included in first-line tests for hyperkalemia workup. It is, in fact, crucial to address diagnosis and treatment quickly, given the risk of hyperkalemia in hospitalized patients [6]. However, healthcare providers often prescribe expensive tests and medications (i.e., newer potassium binders) to address hyperkalemia with delays in the diagnosis and increase in healthcare expenses.

When approaching patients with cAI and on ACE-is presenting with hyperkalemia, it is important to consider a primary adrenal insufficiency-like picture. The chronic ACTH suppression by exogenous steroid medications can cause adrenal atrophy and subphysiological aldosterone levels in addition to hypocortisolemia. The additive inhibition of the angiotensin-converting enzyme by ACE-is further affects aldosterone production, thus inducing significant hypoaldosteronism.

The limitations of this case report are that, with only one patient, we cannot formulate robust evidence-based guidance on the treatment of type IV RTA in the particular condition of concomitant cAI and ACE-is use. Also, we do not have a documented measure of cortisol and ACTH in our patient at the time of presentation. Last, a combination of medications, such as bactrim and lisinopril, and cAI can explain type IV RTA in this patient; however, the prompt response to fludrocortisone and the absence of side effects on a long-term mineralocorticoid replacement therapy is indicative of a true aldosteronism deficiency.

## 4. Conclusion

In patients with unexplained hyperkalemia and underlying predisposing conditions, type IV RTA must always be considered. In particular, the index of suspicion must be high in patients with cAI and on concomitant ACE-I treatment leading to further suppression of aldosterone activity. Occasionally, in secondary or iatrogenic hypoaldosteronism, refractory hyperkalemia responds only to sodium bicarbonate and fludrocortisone given in combination.

## Figures and Tables

**Table 1:** Hospital course timeline starting from day 30; medication management; serum potassium, bicarbonate, and creatinine levels.

Day of admission	Potassium (mmol/L)	Bicarbonate (mmol/L)	Creatinine (mg/dL)
30	4.0	21.6	1.2
**Lisinopril 10 mg daily was restarted on day 30**			
31	3.6	21.9	1.3
32	3.9	21.3	**1.4**
33	**5.5**	**17.5**	**1.5**
**Lisinopril discontinued due to acute kidney injury on day 33**			
34	**5.9**	**19.1**	**1.7**
**TMP-SMX was switched to doxycycline; the patient received patiromer on day 34**			
35	**6**	**19.6**	**1.4**
35	**6.3**	**19.6**	**1.5**
**Sodium bicarbonate 1300 mg three times a day was started on day 35**			
36	**5.5**	20.5	1.2
**Fludrocortisone 0.1 mg daily was started on day 36**			
37	4.4	20.8	1.1
38	4.5	21.6	1.2
**Discharge**			

Values of serum potassium, serum bicarbonate, and serum creatinine levels over the course of admission and treatment modifications secondary to acute kidney injury, hyperkalemia, and NAGMA. Based on our laboratory normal range, abnormal values are highlighted in bold.

## Data Availability

No data were used to support this study as this is a case report on one patient and information is confidential. Additional data can be made available from the corresponding author upon request.

## Abbreviations

RTA: Renal tubular acidosis
T2DM: Type 2 diabetes mellitus
ACE-is: Angiotensin-converting enzyme inhibitors
TMP-SMX: Trimethoprim sulfamethoxazole
CKD: Chronic kidney disease
cAI: Chronic adrenal insufficiency
MRSA: Methicillin-resistant *Staphylococcus aureus*
NAGMA: Nonanion gap metabolic acidosis
K: Potassium.
